# Supplementary material for: Comparative multiomics analysis of cell physiological state after culture in a basket bioreactor
Source: Sci Rep. 2022 Nov 23;12:20161. doi: 10.1038/s41598-022-24687-4 (PMC9686226; doi:10.1038/s41598-022-24687-4)
Supplement: Supplementary file 1 — Supplementary Information 1. [file 41598_2022_24687_MOESM1_ESM.zip › raw data/Proteomic raw data/6.KEGGenrichment/EG.vs.CG/ALL/pathway/src/00830.html]

map00830


- K11152, K00061, K11151, R03048, R08380"

- Down regulated proteins


A0A0D9RMB0(Description:NADP-retinol dehydrogenase;Gene:RDH10;log2FC:-1.9948)

- K11150, K11151, K11152, K11153, K11154, R02124, R08379"

- Down regulated proteins

A0A0D9RMB0(Description:NADP-retinol dehydrogenase;Gene:RDH10;log2FC:-1.9948)

- K11146, K11147, K11148, K11149, R02124, R08379"

- Down regulated proteins

A0A0D9S8K6(Description:Uncharacterized protein;Gene:DHRS3;log2FC:-1.5074)

Close
